# Selective Pharmacological Modulation of Pyramidal Neurons and Interneurons in the CA1 Region of the Rat Hippocampus

**DOI:** 10.3389/fphar.2013.00024

**Published:** 2013-03-13

**Authors:** Marzia Martina, Tanya Comas, Geoffrey A. R. Mealing

**Affiliations:** ^1^Human Health Therapeutics, National Research Council of CanadaOttawa, ON, Canada

**Keywords:** NMDA receptor, NMDA receptor antagonists, electrophysiology, excitatory transmission, hippocampal neurons, memantine

## Abstract

The hippocampus is a complex network tightly regulated by interactions between excitatory and inhibitory neurons. In neurodegenerative disorders where cognitive functions such as learning and memory are impaired this excitation-inhibition balance may be altered. Interestingly, the uncompetitive *N*-methyl-d-aspartate receptor (NMDAR) antagonist memantine, currently in clinical use for the treatment of Alzheimer’s disease, may alter the excitation-inhibition balance in the hippocampus. However, the specific mechanism by which memantine exerts this action is not clear. To better elucidate the effect of memantine on hippocampal circuitry, we studied its pharmacology on NMDAR currents in both pyramidal cells (PCs) and interneurons (Ints) in the CA1 region of the hippocampus. Applying whole-cell patch-clamp methodology to acute rat hippocampal slices, we report that memantine antagonism is more robust in PCs than in Ints. Using specific NMDAR subunit antagonists, we determined that this selective antagonism of memantine is attributable to specific differences in the molecular make-up of the NMDARs in excitatory and inhibitory neurons. These findings offer new insight into the mechanism of action and therapeutic potential of NMDA receptor pharmacology in modulating hippocampal excitability.

## Introduction

While the pathogenesis of neurodegenerative diseases remains poorly understood, the involvement of the glutamatergic system and, specifically, of the *N*-methyl-d-aspartate receptor (NMDAR) in the pathogenesis of numerous neurodegenerative disorders is widely recognized (Hardingham and Bading, 2010).

*N*-Methyl-d-aspartate receptor are heteromultimeric channels comprised of three different subunit families (NR1, NR2A-D, NR3A-B; Meguro et al., 1992; Monyer et al., 1992; Dingledine et al., 1999). Different combinations of these subunits confer the pharmacological profile, gating properties, and Mg^2+^ sensitivity to the NMDAR complex (Sucher et al., 1995; Danysz and Parsons, 1998). Because of their properties, NMDARs are important to fast synaptic neurotransmission and synaptic plasticity (Cull-Candy et al., 2001). NMDAR activation requires the presence of glutamate and a co-agonist (glycine or d-serine) as well as the relief of the Mg^2+^ block through depolarization (Danysz and Parsons, 1998). Once open, NMDARs allow the passage of Ca^2+^ and, to a lesser extent, Na^+^ and K^+^ (fast synaptic transmission). Ca^2+^ influx through the NMDAR is also responsible for the persistent changes observed in long-term potentiation (LTP), the cellular mechanism underlying synaptic plasticity (Nicoll and Malenka, 1999; Cull-Candy et al., 2001), which is implicated in cognitive functions such as learning and memory.

One of the most devastating symptoms associated with neurodegenerative disorders is cognitive impairment, and damage to the hippocampal formation, the principal region associated with learning and memory, is linked to these disorders. The hippocampus is a complex network that consists of tightly regulated interactions between excitation [glutamatergic dentate granular cells, CA1, and CA3 pyramidal cells (PCs)] and inhibition [GABAergic interneurons (Ints); Woodson et al., 1989]. Inhibitory Ints play a crucial role in regulating the interactions between PCs (Klausberger et al., 2005; Klausberger and Somogyi, 2008; Isaacson and Scanziani, 2011; Kullmann, 2011), including population oscillations, plasticity, epileptic synchronization, hormonal effects, and cortical development. Palop and Mucke (2010) suggested that in Alzheimer’s disease (AD), dysfunction of Ints likely increases synchrony among excitatory principal cells and contributes to the destabilization of neuronal networks. In addition, using animal model studies, it has been hypothesized that in AD, the excitation-inhibition balance in hippocampal neuronal circuitry is shifted, resulting in over-excitation (Schmitt, 2005). Furthermore, memantine, an uncompetitive NMDAR antagonist clinically used for the treatment of mild to severe AD (2003 EU, USA), may restore balance between excitation and inhibition (Schmitt, 2005; Parsons et al., 2007). Very recently, Guadagna et al. (2012) suggested that, in mice, clinically relevant doses of memantine promote neuronal network synchronization in the hippocampus.

To elucidate the mechanisms by which memantine preserves basal synaptic activity and inhibits excitotoxicity in the AD brain, its pharmacology has been studied extensively (for a review see Parsons et al., 2007). Memantine is a use-dependent NMDAR antagonist (open-channel blocker). Its primary binding site overlaps that of Mg^2+^ (Kashiwagi et al., 2002; Chen and Lipton, 2005), it has low-affinity for the NMDAR, it has relatively rapid on-off binding kinetics, and it exhibits partial trapping (Parsons et al., 1995; Mealing et al., 1999). Collectively, these properties can be extrapolated to partially explain memantine’s modulation of hippocampal activity. However, pharmacological studies to date have been conducted using cultured cortical neurons or using NMDARs expressed in cell lines, but have not considered the effect of memantine on native synaptic NMDARs in excitatory and inhibitory neurons incorporated into physiological neuronal networks. To address this limitation, we performed whole-cell patch-clamp on acute rat hippocampal brain slices and studied the effect of memantine on NMDARs in PCs and Ints. We found that memantine antagonism of NMDAR currents was more robust in PCs than in Ints at low concentrations (0.1–1 μM), while it was comparable at concentrations higher than 1 μM. Using pharmacological blockage of specific NMDAR subunits, we found that memantine antagonism is dependent upon on the molecular make-up of the NMDAR that different neuronal types express.

## Materials and Methods

This study was approved by the Animal Care Committee of the National Research Council of Canada.

### Preparation of hippocampal slices

Coronal brain slices containing the hippocampus were obtained from 21- to 28-day-old Sprague–Dawley rats. Prior to decapitation, animals were anesthetized with isofluorane (4%, 2 L/min O_2_ flow rate), in conformity with the guidelines of the Canadian Council of Animal Care. The brain was removed and placed in an oxygenated (95% O_2_/5% CO_2_) physiological solution, artificial cerebrospinal fluid (ACSF), at 4°C containing (mM) 126 NaCl, 2.5 KCl, 1 MgCl_2_, 26 NaHCO_3_, 1.25 NaH_2_PO_4_, 2 CaCl_2_, 1 ascorbic acid, and 10 glucose (300 mOsm, pH = 7.3).

A tissue block containing the hippocampal region of interest was sectioned into 300 μm thick slices using a vibrating microtome (Vibratome Series 300, Vibratome, Bannockburn, IL, USA). Slices were incubated in an oxygenated submersion chamber at room temperature for a minimum of 1 h prior to recording.

### Data recording and analysis

Voltage-clamp experiments were performed at room temperature using borosilicate pipettes filled with a pipette solution containing (in mM) 130 Cs^+^-methanesulfonate, 10 *N*-2-hydroxy-ethylpiperazine- *N*′-2-ethanesulfonic acid (HEPES), 10 CsCl, 2 MgCl_2_, 2 ATP-Mg, 0.2 GTP-tris(hydroxy-methyl)aminomethane, 5 lidocaine *N*-ethylbromide (QX-314), 5 1,2-bis(o-aminophenoxy)ethane-*N*,*N*,*N*′,*N*′-tetraacetic acid; BAPTA). The pH was adjusted to 7.2 with CsOH and osmolarity was adjusted to 280–290 mOsm. The liquid junction potential was measured (∼10 mV) and membrane potential (*V*m) was corrected accordingly. Pipettes had a resistance of 3–5 MΩ. Whole-cell access resistance ranged from 5 to 15 MΩ and was monitored throughout the experiments.

**Figure 1:**
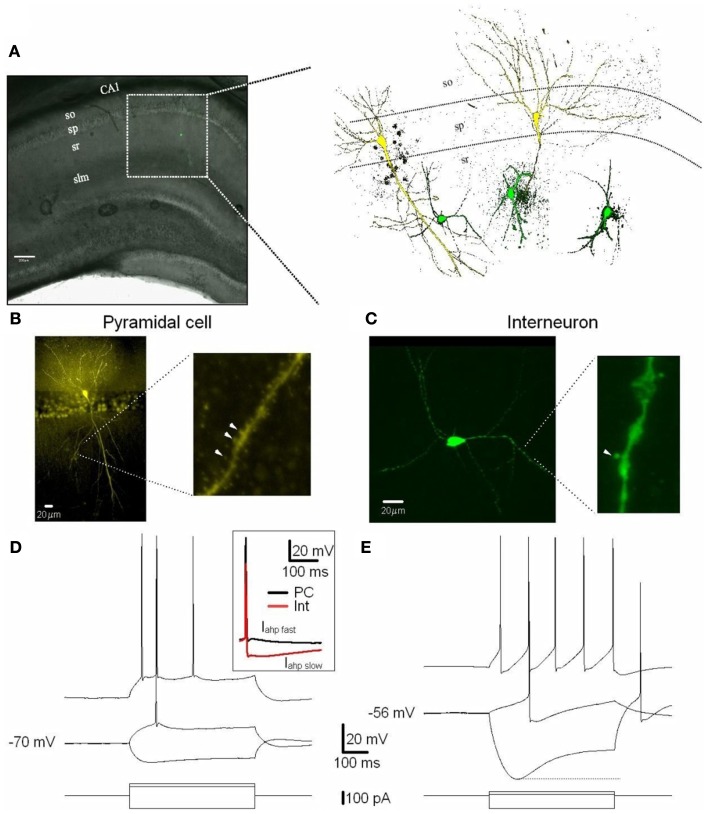
**Excitatory (PCs) and inhibitory (Ints) neurons recorded in the CA1 region of the hippocampus**. **(A)** CA1 PCs and Ints were patch-clamped with a pipette solution containing Lucifer Yellow (2 mM). The location of neurons in the slices was visualized by superimposition of the reflected light image of the hippocampal slice and of the Lucifer yellow fluorescence signal (left panel). The right panel shows reconstructed confocal images of two PCs (yellow) and three Ints (green) recorded in the CA1 sp and sr, respectively. **(B,C)** Confocal images of one PC **(B)** and one Int **(C)** of CA1 pyramidal layer and stratum radiatum, respectively. **(B)** Right and **(C)** right expanded confocal images of dendritic spines. Note the spiny dendritic segment of the PC **(B)** in contrast with the a-spiny **(C)** one of the Int. The arrowheads indicate dendritic spines. **(D,E)** Voltage responses of one PC and one Int (top), to a series of intracellular current pulses (bottom) are shown. The current was applied at rest (−70 and −56 mV for PC and Int, respectively). Inset, action potentials from a PC and Int are superimposed. Note the larger *I*_ahp_ in Int compared to PC. Abbreviations: so, stratum oriens; sp, stratum pyramidale; sr, stratum radiatum; slm, stratus lacunosum moleculare. Scale bar: 200 μm.

Recordings were obtained from PCs and Ints in the stratum pyramidale and stratum radiatum of the CA1 region of the hippocampus, respectively (Figure 1; also see Discussion). We recorded from Ints located ∼250–300 μm from the stratum pyramidale. Following the interneuron classification (Klausberger, 2009), we suggest that the Ints we recorded were Schaffer collateral-associated cells and/or apical dendrite innervating cells which express cholecystokinin (i.e., CCK-expressing Ints; Klausberger, 2009). The soma of Schaffer collateral-associated cells is located mainly in stratum radiatum with dendrites spanning all layers (Figure 1). The axons of these cells innervate the oblique and to a lesser extent basal dendrites of CA1 PCs and Ints mainly in stratum radiatum (Klausberger, 2009). Apical dendrite innervating cells have soma, dendritic, and axonal distributions very similar to those of Schaffer collateral-associated cells (Figure 1). However, electron microscopic investigations have indicated that the apical dendrite-targeting cells innervate preferentially the main apical shaft of CA1 PCs (Klausberger et al., 2005).

Whole-cell patch-clamp recordings were acquired using a Multiclamp 700B amplifier (Molecular Devices, Sunnyvale, CA, USA) under visual control using differential interference contrast and infrared video microscopy (IR-DIC; Olympus BX50WI; Olympus Canada, Inc., Markham, ON, Canada). Whole-cell currents were recorded from individual PCs and Ints voltage-clamped at −70 and −30 mV.

Post-synaptic responses were evoked by electrical stimulation of the Schaffer collaterals with a bipolar microelectrode positioned in the stratum radiatum. Stimulation, consisting of 300 μs uration current pulses (0.1–1 mA; 0.1 Hz), was adjusted to evoke EPSC amplitudes in the range of 60–120 pA at *V*m = −70 mV in ACSF.

To isolate the NMDAR-mediated component of evoked responses we used normal ACSF (at −30 mV) or ACSF containing a low concentration of MgCl_2_ (0.1 mM) with osmolarity maintained by CaCl_2_ (at −70 mV), and the 2-amino-3-(5-methyl-3-oxo-1,2- oxazol-4-yl)propanoic acid receptor (AMPAR) antagonist 1,2,3,4-tetrahydro-6-nitro-2,3-dioxobenzo[f]quinoxaline-7-sulfonamide (NBQX, 20 μM), the GABA_A_ receptor antagonist picrotoxin (50 μM), and the GABA_B_ receptor antagonist 3-[[(3,4-dichlorophenyl)methyl]amino]propyl]diethoxymethyl)phosphinic acid (CGP 52432, 10 μM; Martina et al., 2003).

One pitfall with the patch-clamp technique is the distortion of voltage-gated currents from neurons in brain slices due to non-uniform space-clamp control. To reduce current attenuation due to space-clamp that can occur when recording voltage-gated currents from neurons in brain slices, we recorded the CA1 neurons using a pipette solution containing Cs^2+^ and QX-314, to block K^+^ and Na^2+^ conductances. The additional blockage of AMPAR, GABA_A_ receptor, and the GABA_B_ receptor further reduced space-clamp attenuation. We also tried to obtain recordings with very low series resistances and compensate them as much as possible (90%). Attention was also given to the shape of the evoked responses during off-line analysis (Williams and Mitchell, 2008). Collectively, these measures ensured that space-clamp errors were minimal.

To isolate the AMPAR-mediated component of evoked responses, we used a pipette solution containing (in mM) 130 K^+^-gluconate, 10 HEPES, 10 KCl, 2 MgCl_2_, 2 ATP-Mg, 0.2 GTP, and normal ACSF containing the NMDAR antagonist dl-2-amino-5-phosphonovaleric acid (AP5; 50 μM), picrotoxin (50 μM), and CGP 52432 (10 μM).

A paired-pulse stimulation paradigm was used to evaluate the locus of action of memantine. It is known that alterations in the paired-pulse ratio of evoked post-synaptic currents (EPSCs) following drug application are an indication of a pre-synaptic action of the drug. We delivered two stimuli with an inter-stimuli interval of 100 ms. The paired-pulses were repeated 30 times with an interval of 10 s, and then averaged for analysis. The paired-pulse ratio was determined by dividing the peak amplitude of the second evoked NMDAR current by the peak amplitude of the first (peak 2/peak 1; Thomson, 2000).

Kinetic analysis was performed on averaged evoked EPSCs (30 consecutive traces). The rise-times of NMDAR currents were measured at 10–90%. Their decays were fitted with the exponential functions: *y* = *A*_f_ exp(−*t*/τ_f_) + *A*_s_ exp(−*t*/τ_s_) for double- and *y* = *A*1exp(−*t*/τ) for single exponential decay, where A is the amplitude, τ is the decay time-constant, and the subscript f and s denote fast and slow components, respectively. Weighted time-constants (τ_mean_) were calculated using the equation: τ_mean_ = [*A*_f_/(*A*_f_ + *A*_s_)]τ_f_ + [*A*_s_/(*A*_s_ + *A*_f_)]τ_s_ (Stocca and Vicini, 1998). The dose-response data for memantine on PCs and Ints were fitted with the Hill equation: *Y* = Bottom + [(Top + Bottom)/1 + 10^(LogIC50−X) × HillSlope^]. The variable Bottom is the *Y* value at the bottom plateau (0), Top is the *Y* value at the top of the plateau (100), and LogIC_50_ is the *X* value when the response is halfway between Bottom and Top. The variable HillSlope (Hill coefficient) describes the steepness of the curve. Data were collected using pClamp 10 software (Molecular Devices, Sunnyvale, CA, USA). Analyses were performed off-line with IGOR software (WaveMetrics, Inc., Lake Oswego, OR, USA). Statistical significance of the results was determined with paired Student’s *t*-tests (two-tailed). All values are expressed as means ± SEM, and a *p*-value of <0.05 was considered significant.

**Figure A1:**
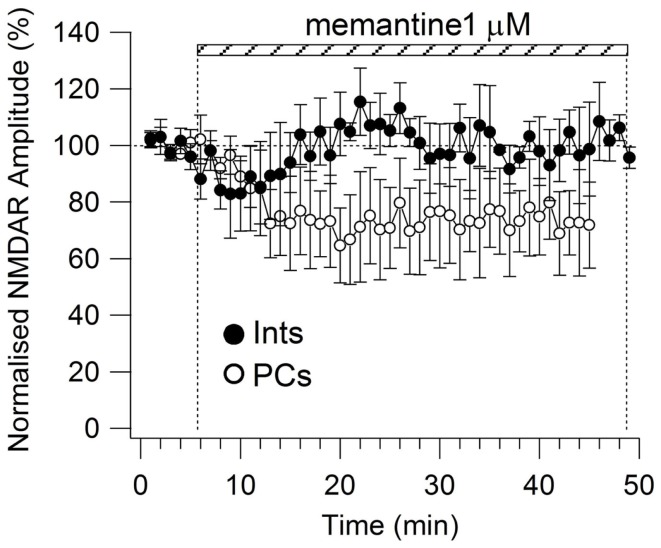
**Time-course of memantine (1 μM)-induced reduction on the NMDAR current amplitude in PCs (empty circle, *n* = 5) and Ints (full circle, *n* = 3), respectively**. Each point (one every min) is the average of six points (stimulations every 10 s). The bars indicate the duration of memantine exposure. Mean ± sem.

All drugs were obtained from Sigma-Aldrich (St Louis, MO, USA), with the exception of NBQX and GCP 52432 which were purchased from Tocris Bioscience (Minneapolis, MN, USA), and NVP-AMM077 which was a kind gift from Dr. Yves Auberson (Novartis, Basel, Switzerland). NVP-AAM077, Ro25-6981, and memantine were applied until the amplitude of the NMDAR currents reached a stable plateau. To establish that a plateau for the effect of memantine was attained (i.e., that equilibrium blockade was achieved), the average responses of two consecutive recordings of 5 min of evoked NMDAR responses needed to display the same amplitude and shape. For every neuron, memantine was applied until a plateau was reached. Longer times were required for low memantine concentrations to attain this plateau (i.e., see Figure A1 in Appendix for 1 μM memantine).

### Electrophysiological and morphological identification of recorded cells

The electro-responsive properties of PCs and Ints were studied in current-clamp by applying 1000 ms current pulses from resting membrane voltage (*V*m) in normal ACSF. Whole-cell recordings were obtained using pipettes filled with a solution containing (mM): 130 K^+^-gluconate, 10 HEPES, 10 KCl, 2 MgCl_2_, 2 ATP, and 0.2 GTP. The amplitude of current pulses was varied in increments of 10 pA. The input resistance (Rin) was estimated in the linear portion of current–voltage plots. The membrane time-constant (τ) was derived from single exponential fits to voltage responses in the linear portion of current–voltage relations. The spike amplitude and the spike amplitude at half-duration were measured from the first action potential evoked by a current pulse.

In some experiments, recorded neurons were identified by including Lucifer Yellow (2 mM) in the pipette. The slices were removed from the chamber and fixed for 1–3 days in 0.1 M phosphate-buffered saline, pH 7.4, containing 4% paraformaldehyde. Slices were washed in dimethyl-sulfoxide (DMSO) for 1 h and visualized with a Fluo-View FV1000 Olympus confocal microscope (Olympus Canada, Inc., Markham, ON, Canada) using 10× and 40× water immersion objectives. Three-dimensional reconstructions of the neurons were made from *z*-series optical sections using the Olympus FV10-ASW Viewer.

## Results

### Physiological and morphological properties of CA1 pyramidal cells and Ints

Cells were visually identified in hippocampal slices with IR-DIC and selected for recordings on the basis of their morphology and localization in specific layers of the CA1 region (see Materials and Methods). PCs and Ints were recorded in the pyramidal layer and stratum radiatum, respectively (Figure 1). A total of 11 Ints and 10 PCs were morphologically identified using Lucifer Yellow (see Materials and Methods). In the experiments designed to record Ints in the stratum radiatum, pipettes were positioned over the ovoid somatic profile. All cells chosen in this way (*n* = 11) were found to be Ints with a-spiny, very sparsely spiny, or varicose dendrites (Figure 1C; Freund and Buzsáki, 1996). Whole-cell recordings were obtained in current-clamp mode to examine the intrinsic membrane properties of PCs (*n* = 10) and Ints (*n* = 11). The PCs generated spike trains that exhibited frequency adaptation when depolarized (Figure 1D). The Ints could sustain high firing rates without or with various degrees of accommodation (Figure 1E). In addition, they had significantly pronounced after-hyperpolarization (*I*_ahp_ _fast_, 10 vs. 2 mV in PCs, *p* = 0.001; *I*_ahp_ _slow_, 14 vs. 3 mV in PCs, *p* = 0.00001; Table 1 and Figure 1D inset) and *I*_h_ current (sag at −100 pA current step: 13.5 ± 1.7 vs. 2.9 ± 0.4 mV in PCs, *p* = 0.0007; Figures 1D,E). A summary of the electrophysiological properties of the PCs and Ints recorded in this study is provided in Table 1. In Ints, resting membrane potential (*V*m) was significantly more depolarized (−57 vs. −69 mV in PCs, *p* = 0.00078), action potential amplitude (spike amplitude) was significantly smaller (69 vs. 96 mV in PCs, *p* = 0.00023), and input resistance (Rin) was significantly larger (457 vs. 96 MΩ in PCs, *p* = 0.0000012), while the duration of the action potential at half-amplitude was not significantly different (1.7 vs. 1.6 ms in PCs, *p* = 0.59). These data are in agreement with that previously reported by Martina et al. (2003).

**Table 1:** **Electrophysiological properties of CA1 PCs and Ints**.

Cell type	Resting membrane potential *V*_m_ (mV)	*R*_in_ (MΩ)	Spike amplitude (mV)	Spike duration at half-amplitude (ms)	τ (ms)	*I*_ahp fast_ (mV)	*I*_ahp slow_ (mV)
PCs *n* = 10	−69.9 ± 0.79	96.6 ± 5.18	96.2 ± 3.33	1.73 ± 0.09	34.9 ± 2.88	1.55 ± 0.51	2.97 ± 0.33
Ints *n* = 11	−56.7 ± 2.72	457 ± 47.58	69.3 ± 3.36	1.65 ± 0.11	28.8 ± 3.23	9.98 ± 1.68	13.9 ± 1.19

### Effect of memantine on NMDAR currents in PCs and Ints

**Figure 2:**
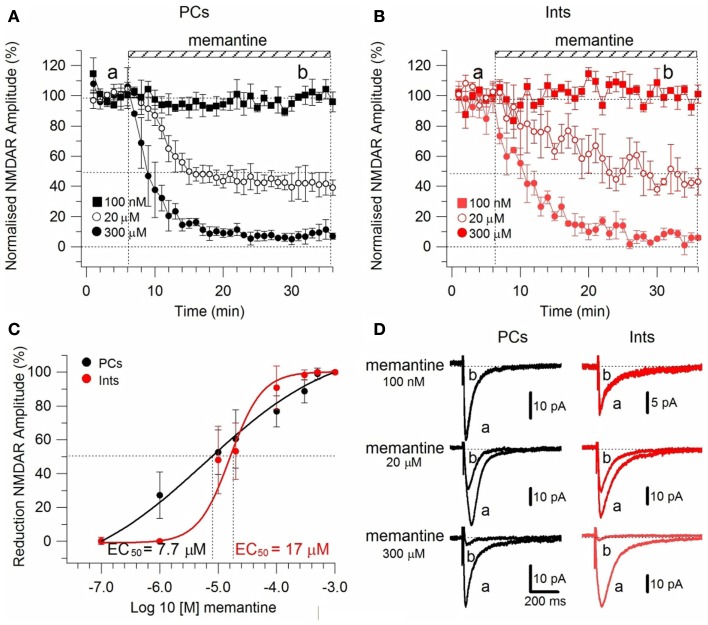
**Effect of memantine on NMDAR currents in PCs and Ints in the CA1 region of the hippocampus**. NMDAR currents were recorded from individual PCs and Ints voltage-clamped at *V*m = −70 mV. **(A,B)** Time-course of memantine-induced reduction on the NMDAR current amplitude in PCs and Ints, respectively. In PCs, bath application of 100 nM, 1 μM, 10 μM, 20 μM, 100 μM, 300 μM, 500 μM, and 1 mM memantine reduced the amplitude of the NMDAR currents by 0% (*n* = 3), 27.28 ± 13.26% (*n* = 5), 52.66 ± 3.52% (*n* = 14), 60.55 ± 2.93% (*n* = 7), 73.82 ± 3.48% (*n* = 7), 87.8 ± 2.42% (*n* = 8), 98.04 ± 1.69% (*n* = 3), and 100% (*n* = 3), respectively. In Ints, bath application of 100 nM, 1 μM, 10 μM, 20 μM, 100 μM, 300 μM, 500 μM memantine reduced the amplitude of NMDAR currents by 0% (*n* = 3), 0.5 ± 0.4% (*n* = 7); 48.3 ± 4.98% (*n* = 16), 53.22 ± 8.35% (*n* = 5), 90.85 ± 6.4% (*n* = 4), 98.29 ± 1.7% (*n* = 3), and 100% (*n* = 3), respectively. Normalized NMDAR current amplitudes (%) are plotted as a function of time for memantine concentrations of 100 nM (full square), 20 μM (empty circle), 300 μM (full circle). Each point (one every min) is the average of six points (stimulations every 10 s). The bars indicate the duration of memantine exposure. Mean ± sem. **(C)** Memantine dose-response curve in PCs (black) and Ints (red), respectively. Error bars represent SD. **(D)** Examples of traces of NMDAR currents measured before (a) and after memantine applications (b) are shown for PCs (black) and Ints (red).

To test the effect of memantine on neurons regulating excitation-inhibition balance in the hippocampal neuronal circuitry, we recorded NMDAR currents in PCs and Ints (Figure 2), and measured the effect of the bath application of memantine at different concentrations. To evoke post-synaptic glutamatergic currents in PCs and Ints, the Shaffer collaterals were stimulated with a bipolar electrode. The NMDAR-mediated component of the post-synaptic current was pharmacologically isolated at *V*m = −70 mV in a low Mg^2+^ACSF (see Materials and Methods). The isolated NMDAR currents displayed an averaged amplitude of 57.46 ± 9.10 pA (*n* = 62) in PCs and 28.00 ± 2.65 pA (*n* = 54) in Ints. Memantine attenuated NMDAR current amplitudes in both PCs (Figure 2A) and Ints (Figure 2B); see Figure 2 legend for percentages of reduction). Memantine antagonism was concentration-dependent, with IC_50_ values of (LogIC_50_ = −5.11) 7.7 μM and (LogIC_50_ = −4.77) 17 μM for PCs and Ints, respectively (Figure 2C*)*. The two IC_50_ values were significantly different (*p* = 0.01326; non-parametric Wilcoxon Rank Test). In addition to the higher affinity of memantine for NMDARs in PCs vs. Ints, the fitting parameters of the Hill equation showed significantly different Hill coefficients (see Materials and Methods) in PCs (Hill coefficient = 0.6101 ± 0.07) and Ints (Hill coefficient = 1.106 ± 0.16; *p* = 0.0086; Figure 2C). A Hill coefficient lower than one indicates positive cooperativity, suggesting that the PCs may possess at least two subtypes of NMDAR, one of which appears to have a higher affinity for memantine, whereas Ints (negative cooperativity) may express a single NMDA receptor subtype with a lower affinity for memantine.

**Figure A2:**
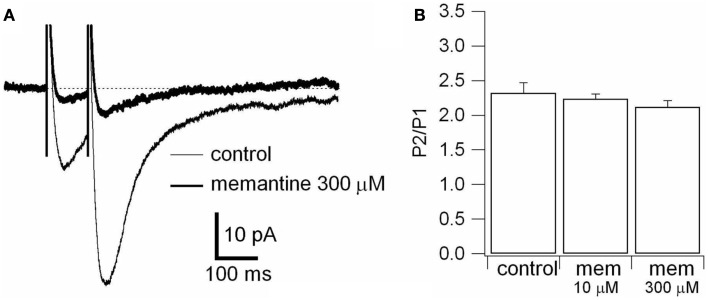
**Effect of memantine on paired-pulse stimulation**. **(A)** Responses were evoked by bipolar electrical stimuli at *V*m = −70 mV in PCs. Two pulses of identical intensity were delivered with an interval of 100 ms. NMDAR evoked responses observed in control (thin line) and during application of memantine (300 μM; thick line) are superimposed. Each trace is an average of 30 traces. **(B)** Histogram of the ratio between the amplitude of the second (P2) and the first (P1) evoked response in absence (control) and in presence of memantine (10 and 300 μM), is shown. The differences were not statistically significant (see text).

To rule out the possibility that these differences were attributable to memantine-mediated changes in neurotransmitter release, we recorded NMDAR currents in PCs and delivered paired-pulse stimulation (100 ms) to the Schaffer collaterals in the absence and presence of memantine (10 and 300 μM; see Materials and Methods). In the absence and presence of memantine (10 and 300 μM) the second response showed facilitation. The evoked currents had peak 2/peak 1 ratios of 2.32 ± 0.15 (*n* = 8) in the absence of memantine and did not change in presence of memantine at 10 μM 2.24 ± 0.07 (*n* = 4) and 300 μM 2.12 ± 0.09 (*n* = 4), respectively (Figure A2 in Appendix). These values were not significantly different (*p* = 0.69 and *p* = 0.36 for 10 and 300 μM memantine, respectively), demonstrating that memantine does not alter neurotransmitter release by acting on pre-synaptic afferents.

**Figure A3:**
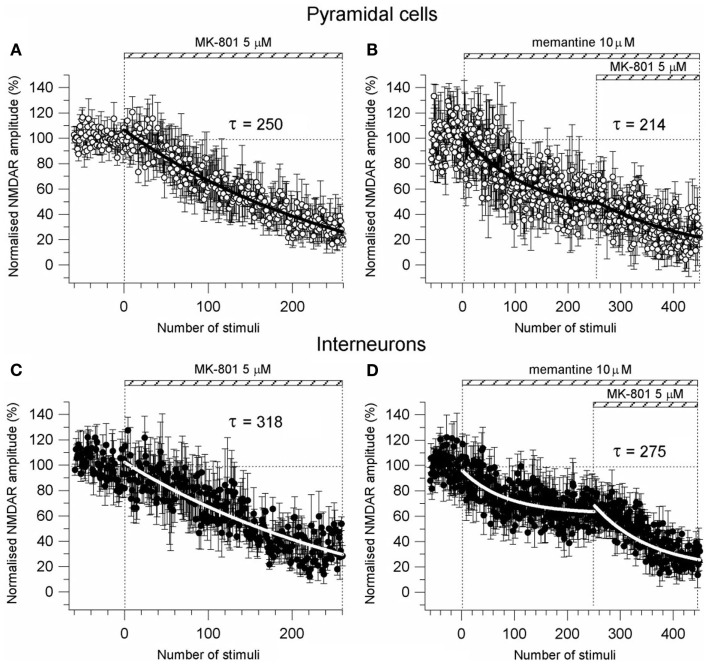
**Effect of memantine on pre-synaptic probability of release**. **(A**–**C)** Time-course of MK-801 (5 μM) induced reduction of the NMDAR current amplitude in PCs (*n* = 8) and Ints (*n* = 7), respectively. NMDAR currents were recorded from individual PCs and Ints voltage-clamped at *V*m = −70 mV. **(B,D)** Time-course of MK-801 (5 μM) + memantine (10 μM) induced reduction of the NMDAR current amplitude in PCs (*n* = 4) and Ints (*n* = 8), respectively. Normalized NMDAR current amplitudes (%) are plotted as a function of number of stimuli. NMDAR responses were evoked every 5 s. The bars indicate the duration of drug application. The time-courses were obtained by fitting the curves with a single exponential [*y* = *A*1exp(−*t*/τ)] (solid bold lines).

To further explore the possibility of a memantine effect on neurotransmitter release we used the NMDA open-channel blocker MK-801. Rosenmund et al. (1993) showed that the rate of progressive MK-801 block of NMDAR currents reflects the probability of pre-synaptic neurotransmitter release. If the probability of release is high, more terminals will release neurotransmitter, more post-synaptic NMDAR channels will open, and the MK-801 progressive block will be more rapid. Consistent with this hypothesis, we measured the progressive MK-801 (5 μM) block of NMDAR currents before and after application of memantine (10 μM) in PCs and Ints to estimate the effect of memantine on the probability of pre-synaptic release. NMDAR currents were evoked every 5 s and MK-801 time-course measured by the fitting of a single exponential [*y* = *A*1exp(−*t*/τ)] of the NMDAR current peak amplitude plotted against stimulation number (Figure A3 in Appendix). The MK-801 block rate was 250 stimuli in PCs (*n* = 8) and 318 stimuli in Ints (*n* = 7), suggesting a higher probability of release in PCs compared to Ints (Figure A3 in Appendix). This is not surprising considering that the non-uniform size and structure of synaptic terminals in the central nervous system results in variable probabilities of release at terminals within a single synapse, and is not the same for different terminals on different types of neurons. Next, memantine (10 μM) was applied until NMDAR currents attenuated to a plateau, then MK-801 was co-applied (Figure A3 in Appendix). The block rate of MK-801 + memantine was 214 stimuli in PCs (*n* = 4) and 275 stimuli in Ints (*n* = 8). These values were not significantly different from those obtained with MK-801 alone (PCs: *p* = 0.35; Ints: *p* = 0.91). These results support the previous finding that memantine had no effect on pre-synaptic release probability.

**Figure A4:**
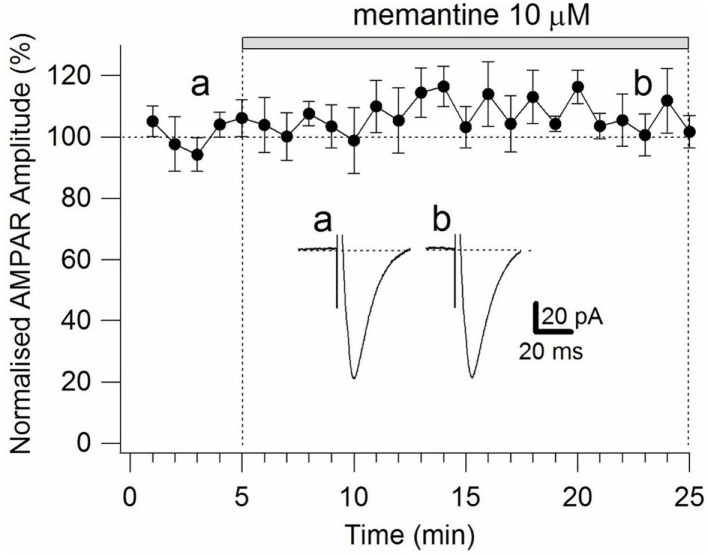
**Time-course of the effects of memantine on AMPAR currents in PCs**. AMPAR currents were recorded from individual PCs voltage-clamped at *V*m = −70 mV. Normalized AMPAR current amplitudes (%) are plotted as a function of time. Each point (one every min) is the average of six points (stimulations every 10 s). The bars indicate the duration of memantine exposure. Examples of traces of AMPAR currents measured before (a) and after memantine (b) application are shown for a PC. Each trace is an average of 30 acquisitions.

To ensure that the observed effects of memantine were specific to NMDARs, we evoked AMPAR currents in the absence and presence of memantine (10 μM; see Materials and Methods). Evoked AMPAR currents had an activation time-constant of 2.47 ± 0.73 ms and a deactivation kinetic of 20.18 ± 2.7 ms (*n* = 4). After 25 min of continuous memantine exposure, the activation time-constant (2.38 ± 0.54 ms), the deactivation kinetic (21.60 ± 2.7 ms, *n* = 4), and the current amplitude (51.42 ± 13.33 pA control, 53.75 ± 12.6 pA memantine, *n* = 4; Figure A4 in Appendix) did not change significantly (*p* = 0.9). These observations indicate that memantine’s effects were limited to NMDAR currents on Schaffer collateral-CA1 synapses.

The primary binding site of memantine on NMDARs overlaps that of Mg^2+^ (Kashiwagi et al., 2002; Chen and Lipton, 2005), however, memantine is thought to act as a more effective surrogate for the divalent Mg^2+^ (Parsons et al., 2007). In the hippocampal network, the Shaffer collaterals release glutamate that generates post-synaptic currents (PSCs), which depend largely on the activation of AMPARs. AMPARs depolarize the post-synaptic membrane causing the Mg^2+^ block to be relieved and NMDARs to open.

**Figure 3:**
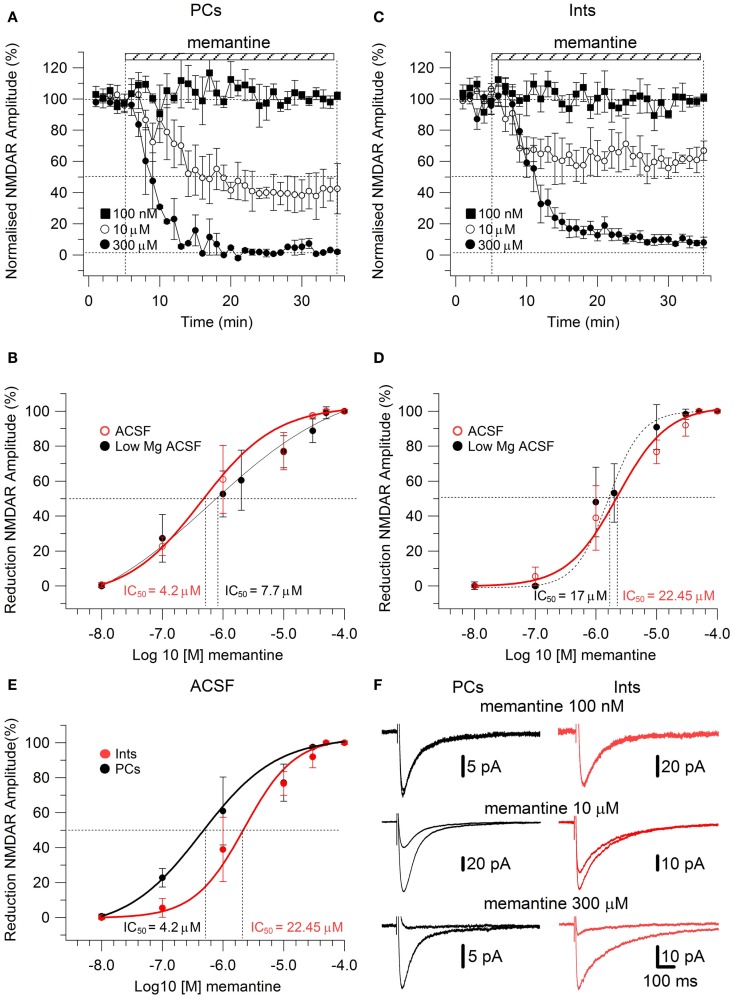
**Effect of memantine on NMDAR currents recorded in presence of Mg^2+^ at V_m_ = −30, in PCs and Ints in the CA1 region of the hippocampus**. **(A–C)** Time-course of memantine-induced reduction on the NMDAR current amplitude in PCs and Ints, respectively. Normalized NMDAR current amplitudes (%) are plotted as a function of time for memantine concentrations of 100 nM (full square), 10 μM (empty circle), 300 μM (full circle). Each point (one every min) is the average of six points (stimulations every 10 s). The bars indicate the duration of memantine exposure. Mean ± sem. **(B,D)** Memantine dose-response curve for PCs **(B)** and Ints **(D)** in absence (full black circle; same data as in Figure 2C) and presence (empty red circle) of physiological concentration of Mg^2+^ respectively. **(E)** Summary of the memantine dose-response curve in PCs (black) and Ints (red), in normal ACSF. Error bars represent SD. **(F)** Examples of traces of NMDAR currents measured before and after memantine applications are shown for PCs (black) and Ints (red).

To study the effect of memantine on NMDAR currents in PCs and Ints in physiological Mg^2+^, we recorded NMDAR-mediated component of the post-synaptic current as described above but in normal ACSF (see also Materials and Methods). The neurons were held at −30 mV to release the Mg^2+^ block and memantine was added to the bath solution at different concentrations. The cells were depolarized to −30 mV since, in the NMDAR IV curve, the larger inward NMDAR currents are evoked at this potential (Nowak et al., 1984). As in low Mg^2+^ ACSF, memantine attenuated NMDAR current amplitudes in both PCs and Ints (Figure 3). In PCs, bath application of 100 nM, 1 μM, 10 μM, 100 μM, 300 μM, 500 μM, and 1 mM memantine reduced the amplitude of the NMDAR currents 0.7 ± 1.15% (*n* = 4), 22.73 ± 2.66% (*n* = 4), 60.97 ± 9.72% (*n* = 4), 77.15 ± 4.77% (*n* = 5), 97.52 ± 0.63% (*n* = 2), 100% (*n* = 3), and 100% (*n* = 3), respectively. In Ints, bath application of 100 nM, 1 μM, 10 μM, 100 μM, 300 μM, 500 μM, and 1 mM memantine reduced the amplitude of the NMDAR currents 0% (*n* = 3), 5.48 ± 3.09% (*n* = 4); 38.97 ± 6.96% (*n* = 7), 76.70 ± 3.38% (*n* = 4), 91.99 ± 2.80% (*n* = 5), 100% (*n* = 3); 100% (*n* = 3). As in low Mg^2+^ ACSF, memantine antagonism was concentration-dependent in both PCs and Ints. At −30 mV in presence of Mg^2+^, IC_50_ values were (LogIC_50_ = −5.37) 4.2 μM and (LogIC_50_ = −4.64) 22.45 μM for PCs and Ints, respectively, further supporting a higher affinity of memantine for PCs’ NMDAR currents. The two IC_50_ values were significantly different (*p* = 0.000232; non-parametric Wilcoxon Rank Test).

In addition to the higher affinity of memantine for NMDARs in PCs vs. Ints, the fitting parameters of the Hill equation showed significantly different Hill coefficients (see Materials and Methods) in PCs (Hill coefficient = 0.617 ± 0.02) and Ints (Hill coefficient = 0.879 ± 0.08; *p* = 0.003; Figure 3). Hill coefficients lower than one indicate positive cooperativity, suggesting that, in the virtual absence of Mg^2+^, both PCs and Ints possess at least two subtypes of NMDAR; in contrast, the Hill coefficient values obtained in presence of Mg^2+^ suggested that Ints (negative cooperativity) may express a single NMDA receptor subtype with a lower affinity for memantine.

### Molecular make-up of NMDARs in PCs and Ints

**Figure 4:**
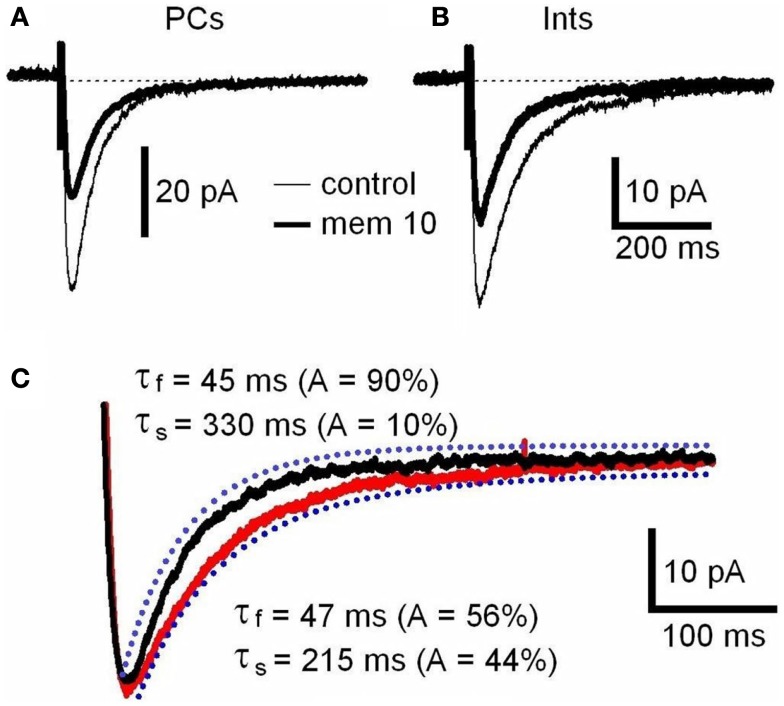
**NMDAR deactivation kinetics in PCs and Ints**. **(A,B)** Examples of traces of NMDAR currents measured before (control, thin line) and after memantine (10 μM; thick line) application are shown for PCs and Ints, respectively. NMDAR currents were recorded from individual PCs and Ints voltage-clamped at *V*m = −70 mV. Each trace is an average of 30 acquisitions. **(C)** Decay time-course of NMDAR currents recorded in PCs (black line) and Ints (red line). The traces are normalized to the amplitude. Blue dotted line: fitting of the deactivation kinetics of NMDAR currents. Note that the decay of the NMDAR currents in Ints (red line) was slower than that of PCs (black line).

*N*-Methyl-d-aspartate receptor are composed of NR1 and NR2 subunits. The expression of NR1/NR2A subunits produces channels with faster deactivation (tens of milliseconds) than NR1/NR2B or NR1/NR2C (hundreds of milliseconds; Stocca and Vicini, 1998), whereas the NMDARs containing NR1/NR2D subunits display very slow kinetics (seconds; Stocca and Vicini, 1998; Misra et al., 2000). In the hippocampus PCs express mainly NR2A and NR2B subunits, while Ints have a larger percentage of NR2C and NR2D subunits (Monyer et al., 1994; Martina et al., 2003). To verify the difference in subunit composition in the two types of neurons, we observed the biophysical properties of the NMDAR currents in PCs and Ints. The NMDAR current decay in both PCs and Ints was best-fitted with a bi-exponential function (Figure 4). The best-fitted decay values for PCs and Ints are shown in Table 2. The values of τ_deact f_ and τ_deact s_ between PCs and Ints were not significantly different. However, the difference in relative portions (*A*_f_ and *A*_s_) of decay time-constants in PCs and Ints was significant and hence the τ_mean_ (Table 2; see Materials and Methods), confirming the different molecular make-up of NMDARs in PCs and Ints.

**Table 2:** **Decay time-constants of the NMDAR currents in PCs and Ints**.

	τ_deact f_ (ms)	τ_deact s_ (ms)	*A*_f_ (%)	*A*_s_ (%)	τ_mean_ (ms)
PCs *n* = 62	54.75 ± 2.02	493 ± 57.76	79.36 ± 1.97	20.64 ± 1.97	130.6 ± 11.80
Ints *n* = 54	69.14 ± 9.26	470 ± 73.50	57.95 ± 3.63*	42.05 ± 3.63*	206.6 ± 25.05*

Values are means ± sem. Electrically evoked NMDAR currents were recorded in low Mg^2+^ ACSF (0.1 mM) in the presence of picrotoxin (50 μM), NBQX (20 μM), and CGP 52432 (10 μM). The fast and the slow components of the deactivation kinetics are designed by f and s, respectively. *Indicates a significant difference between the indicated values and their controls (*A*_f_, *p* = 6.3e−08; *A*_s_, *p* = 6.3e−08; τ_mean_, *p* = 0.0016).

### Effect of memantine on NVP-AAM077- and Ro25-6981-resistant NMDAR currents in PCs and Ints

To determine the effect of memantine on different components of NMDAR currents, we recorded currents in presence of the NR2B-containing NMDARs antagonist Ro25-6981 (500 nM; Fischer et al., 1997) and the NR2A-containing NMDARs antagonist NVP-AAM077 (50 nM; Auberson et al., 2002; Weitlauf et al., 2005). NMDAR currents were evoked every 10 s by releasing pre-synaptic glutamate via electrical stimulation and the antagonists were added to the bath solution via perfusion (see above).

Ro25-6981 is a potent and selective activity-dependent blocker of NMDARs containing the NR2B subunit. IC_50_ values are 0.009 and 52 μM for cloned receptor subunit combinations NR1C/NR2B and NR1C/NR2A, respectively (Fischer et al., 1997). However, NR2B antagonists are influenced by Mg^2+^ concentration and NR2B-directed NMDAR antagonists in the hippocampal slice are more potent in the absence of Mg^2+^ (Williams, 1993; Mott et al., 1998; Rauner and Köhr, 2011). Consequently, in this experimental series, NMDAR currents were recorded in the virtual absence of Mg^2+^ (low Mg^2+^ ACSF). Under these conditions, Ro25-6981 (500 nM) reduced the amplitude of the NMDAR currents by 24.45 ± 6.71% (*n* = 9) in PCs and by 35.07 ± 6.98% (*n* = 8) in Ints, respectively (Figure 6A).

NVP-AAM77 is a competitive antagonist of NR2A-containing NMDARs. It has been shown that for glutamate-evoked currents, the IC_50_ values for NVP-AAM077 acting at NR1/NR2A and NR1/NR2B NMDA receptors were 16 ± 2 nM (NR1/NR2A) and 302 ± 18 nM (NR1/NR2B; Frizelle et al., 2006), respectively. NVP-AAM077 (50 nM) reduced the amplitude of the NMDAR currents by 45.5 ± 3.40% (*n* = 7) in PCs and by 21.00 ± 9.49% (*n* = 7) in Ints, respectively. However, caution is warranted when interpreting NVP-AAM077 data since NVP-AAM077 (400 nM) has been reported to target non-NR2A-containing NMDARs when applied 30 min prior to an agonist (Weitlauf et al., 2005). However, if antagonism of NVP-AAM077 on NMDAR currents is similar at 50 and 400 nM, then the specificity of NVP-AAM077 for NR2A was conserved under the experimental conditions employed here. Bath application of NVP-AAM077 (400 nM) reduced the amplitude of the NMDAR currents by 50.54 ± 3.90% (*n* = 15) in PCs and by 21.50 ± 16.3% (*n* = 8) in Ints, respectively. The plateau effect of NVP-AAM077 at 50 and at 400 nM was reached after 10 min of perfusion (see Figure 6A) and remained constant, indicating that NVP-AAM077’s specificity for NR2A was conserved even at 400 nM. The reductions in current caused by NVP-AAM077 (400 nM) and (50 nM) were not significantly different (*p* = 0.223 for PCs and *p* = 0.428 for Ints), suggesting that only NR2A-containing NMDARs were antagonized by NVP-AAM077.

The difference in the percentage of NMDARs containing the NR2A and NR2B subunit in PCs and Ints (Monyer et al., 1994; Martina et al., 2003) can also account for the faster deactivation decay observed in PCs (Figure 4C; see Discussion).

**Figure 5:**
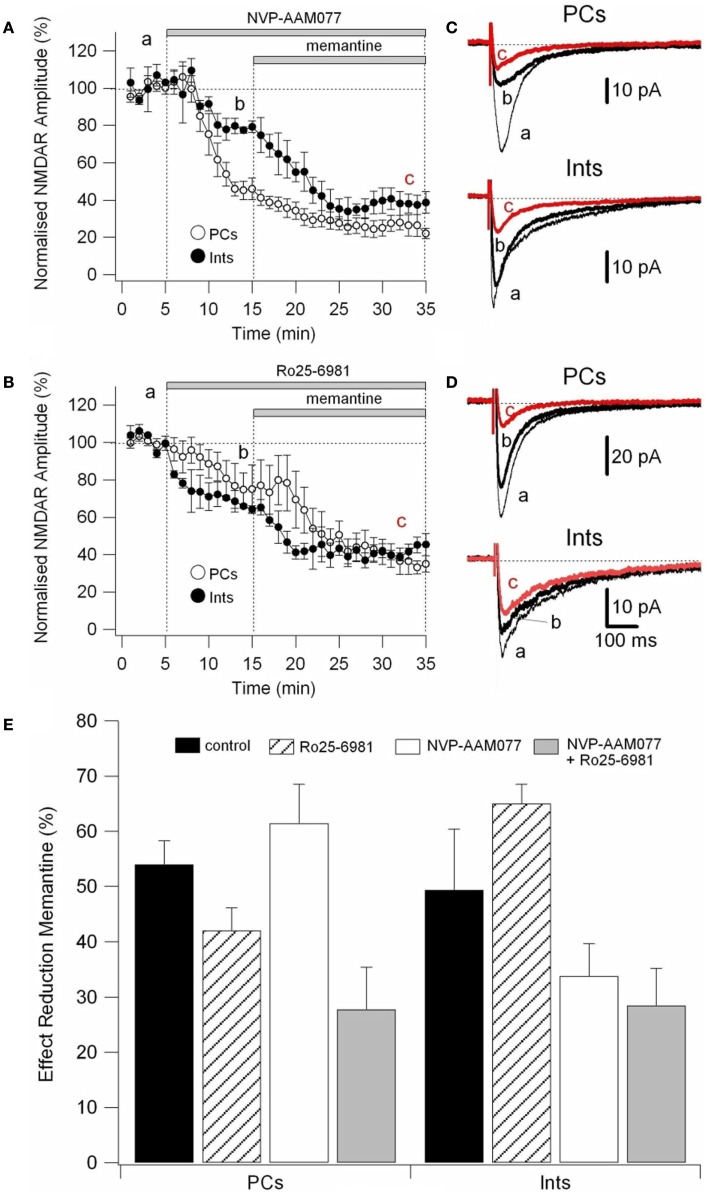
**Effect of memantine on NVP-AAM077- and Ro25-6981-resistent NMDAR currents in PCs and Ints**. NMDAR currents were recorded from individual PCs and Ints voltage-clamped at *V*m = −70 mV. **(A,B)** Time-course of the NVP-AAM077, Ro25-6981 memantine-induced reduction on NMDAR current amplitude in PCs (empty circle) and Ints (full circle). Normalized NMDAR current amplitudes (%) are plotted as a function of time. Each point (one every min) is the average of six points (stimulation every 10 s). The bars indicate the duration of drug application. **(C,D)** Examples of traces of NMDAR currents recorded in PCs (top) and Ints (bottom). Each trace is an average of 30 traces. **(E)** Histogram showing the effect of memantine (10 μM) in PCs and Ints in control (black), in presence of Ro25-6981 (dashed lines), in presence of NVP-AMM077 (white), and in presence of NVP-AMM077 + Ro25-6981 (gray). The magnitude of the memantine effect was measured after 25 min of exposure.

We then applied memantine and measured its effect on different components of NMDARs (Figure 5E). Memantine was used at 10 μM since this concentration was close to the IC_50_ for PCs and Ints.

In the presence of NVP-AAM077, the residual NMDAR currents (see decay time-constants in Tables 3 and 4) were reduced by memantine by 42.02 ± 4.13% (*n* = 15; in the presence of 400 nM NVP-AAM077) and 42.90 ± 3.54% (*n* = 7; in the presence of 50 nM NVP-AAM077) in PCs and by 64.76 ± 2.76% (*n* = 8 in the presence of 400 nM NVP-AAM077) and 58.00 ± 9.96% (*n* = 5; in the presence of 50 nM NVP-AAM077) Ints, respectively (Figures 5A–D). The values for NVP-AAM077 used at 400 and 50 nM were not significantly different (*p* = 0.442 for PCs and *p* = 0.258 for Ints), while the differences between PCs and Ints were (400 nM: *p* = 0.026; 50 nM: *p* = 0.039).

**Table 3:** **Decay time-constants of the NVP-AAM077-, Ro25-6981, and NVP-AAM077 + Ro-6981-resistant NMDAR currents in PCs**.

	τ_deact f_ (ms)	τ_deact s_ (ms)	*A*_f_ (%)	*A*_s_ (%)	τ_mean_ (ms)
NVP-AAM077 *n* = 18	74.27 ± 5.17	514 ± 86.69	70.56 ± 1.87	29.44 ± 1.97	187.6 ± 5.09
Ro25-6981 *n* = 9	60.84 ± 5.65	382 ± 40.32	86.88 ± 2.25	13.12 ± 2.25	97.3 ± 6.89*
NVP-AAM077 and Ro25-6981 *n* = 4	84.78 ± 5.56	592 ± 65.04	70.56 ± 3.12	29.43 ± 3.12	237.3 ± 2.45*

Values are means ± sem. Electrically evoked NMDAR currents were recorded in low Mg^2+^ ACSF (0.1 mM) in the presence of picrotoxin (50 μM), NBQX (20 μM) and CGP 52432 (10 μM). The fast and the slow components of the deactivation kinetics are designated by f and s, respectively. *Indicates a significant difference between the indicate values and their controls (τ_mean_ Ro25-6981 vs. NVP-AAM077, *p* = 0.002; τ_mean_ NVP-AAM077 + Ro25-6981 vs. NVP-AAM077, *p* = 0.002).

**Table 4:** **Decay time-constants of the NVP-AAM077-, Ro25-6981, and NVP-AAM077 + Ro-6981-resistent NMDAR currents in Ints**.

	τ_deact f_ (ms)	τ_deact s_ (ms)	*A*_f_ (%)	*A*_s_ (%)	τ_mean_ (ms)
NVP-AAM077 *n* = 8	77.70 ± 17.7	422 ± 43.55	56.08 ± 6.25	43.92 ± 6.25	233.5 ± 36.5
Ro25-6981 *n* = 9	79.6 ± 21.73	564 ± 58.50	60.23 ± 8.52	39.72 ± 8.52	237.1 ± 34.04
NVP-AAM077 and Ro25-6981 *n* = 8	81.53 ± 19.9	542 ± 48.71	68.7 ± 6.94	31.31 ± 6.94	239.2 ± 22.35

*Values are means ± sem. Electrically evoked NMDAR currents were recorded in low Mg^2+^ ACSF (0.1 mM) in the presence of picrotoxin (50 μM), NBQX (20 μM), and CGP 52432 (10 μM). The fast and slow components of the deactivation kinetics are designated by f and s, respectively*.

In the presence of Ro25-6981, the residual NMDAR currents (see decay time-constants in Tables 3 and 4) were reduced by memantine by 61.49 ± 7.04% (*n* = 9) and 33.77 ± 5.91% (*n* = 8) in PCs and Ints, respectively (Figures 5B,C). The difference between PCs and Ints was significantly different (*p* = 0.0047).

**Figure 6:**
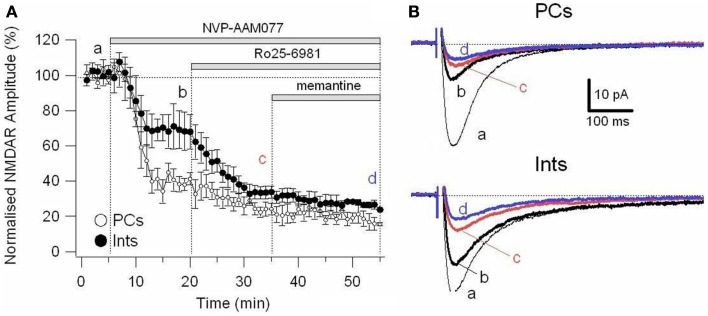
**Effect of memantine on NMDAR currents in PCs and Ints resistant to co-application of NVP-AAM077 and Ro25-6981**. NMDAR currents were recorded from individual PCs and Ints voltage-clamped at *V*m = −70 mV. **(A)** Time-course of the NVP-AAM077, Ro25-6981 memantine-induced reduction on NMDAR current amplitude in PCs (empty circle) and Ints (full circle). Normalized NMDAR current amplitudes (%) are plotted as a function of time. Each point (one every min) is the average of 6 points (stimulation every 10 s). The bars indicate the duration of drug application. **(B)** Examples of traces of NMDAR currents recoded in PCs (top) and Ints (bottom). Each trace is an average of 30 traces.

To further explore the specific effects of memantine on the NR2C and 2D subunit component of NMDARs in PCs and Ints, we co-applied NVP-AAM077 and Ro25-6981. This reduced NMDAR current amplitudes by 74.81 ± 3.11% (*n* = 4) in PCs and 62.18 ± 4.16% (*n* = 8) in Ints (Figure 6). The subsequent application of memantine (10 μM) further reduced the residual NMDAR currents by 27.76 ± 7.67% (*n* = 4; *p* = 0.0429) and 28.43 ± 6.79% (*n* = 8; *p* = 0.018) in PCs and in Ints, respectively (Figures 5C and 6).

## Discussion

**Figure 7:**
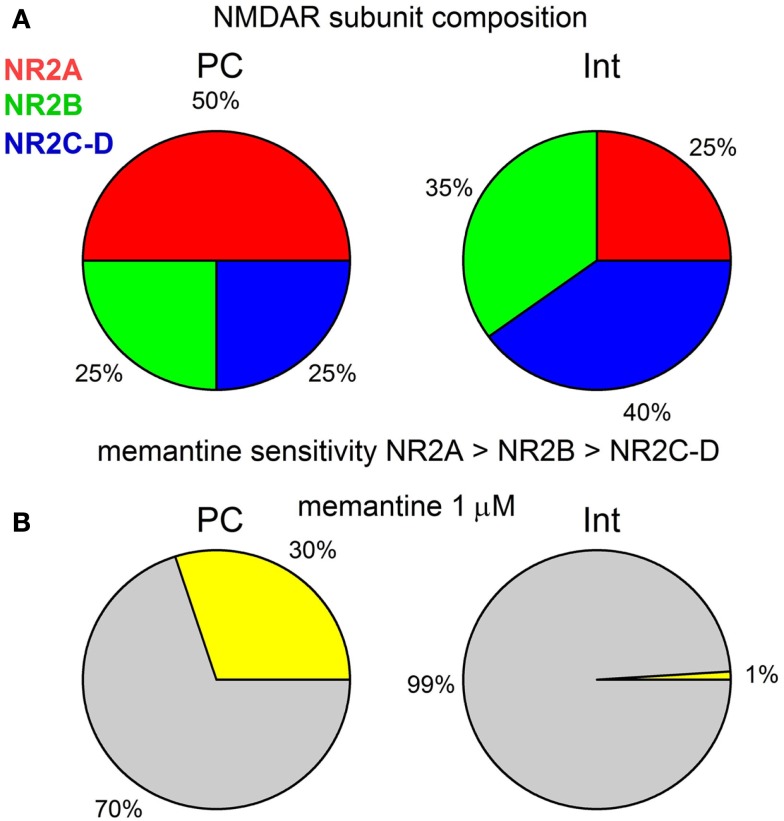
**(A)** Graphical description of the NMDAR subunit composition of PCs and Ints in the CA1 region of the rat hippocampus. **(B)** Graphical representation of the effect of 1 μM memantine on the NMDAR currents in PCs and Ints. The percentage of NMDAR currents blocked by memantine is represented in yellow. Note that for clarity the values are shown without standard deviation.

Our results show that in the CA1 region of the rat hippocampus, memantine antagonism on synaptically evoked NMDAR currents is more robust in PCs than in Ints. We report that this selective antagonism is attributable to specific differences in the molecular make-up of the NMDARs in excitatory and inhibitory neurons (Figure 7).

A CA1 PC receives about 30000 synaptic inputs. The cell body integrates inputs from the dendrites and receives only GABAergic synapses, as does the axon-initial segment, which contributes to action potential generation. The small, oblique dendrites emerging from one or two large apical dendrites and the basal dendrites receive glutamatergic inputs mainly from the hippocampal CA3 area, local axon collaterals, and the amygdala. The apical dendritic tuft is innervated mainly by glutamatergic inputs from the entorhinal cortex and the thalamus. All dendrites also receive local GABAergic inputs from Ints. Such a compartmentalized structure of PCs allows spatially segregated activities at the same time (Klausberger and Somogyi, 2008).

Multiple subtypes of Ints have been described in the hippocampus (Freund and Buzsáki, 1996; Klausberger, 2009; Kullmann, 2011). In this study, recordings were obtained from PCs and Ints in the stratum pyramidale and stratum radiatum of the CA1 region of the hippocampus, respectively (Figure 1). We recorded from Ints in the stratum radiatum of the CA1 region because of their well-described morphology, electrophysiological characteristics, and connectivity (Freund and Buzsáki, 1996; Morin et al., 1996; Parra et al., 1998). Following the interneuron classification (Klausberger, 2009), we suggest that the Ints we recorded were Schaffer collateral-associated cells and/or apical dendrite innervating cells which express cholecystokinin (i.e., CCK-expressing Ints; Klausberger, 2009). It has been hypothesized that CCK-expressing Ints firing during ripple and theta oscillations might indicate a role in shaping the activity of subgroups and assemblies of PCs instead of synchronizing the entire network (Klausberger et al., 2005).

CA1 hippocampal Ints receive two types of excitatory inputs: feedback and feed-forward (Schwartzkroin and Mathers, 1978; Lacaille et al., 1987; Kullmann, 2011), depending on their subtype and location. Notably, in the stratum radiatum of the CA1, glutamatergic inputs to Ints are predominantly from local collaterals of PCs or Schaffer collateral fibers (Freund and Buzsáki, 1996; Kullmann, 2011). The CA3 input to CA1 PCs, mediated by the Schaffer collaterals, is glutamatergic (Amaral and Witter, 1989). Ints that appear to be specialized to mediate feed-forward signaling are often referred to by the identity of the afferent projection that excites them or by the location of their cell bodies. This nomenclature may conceal considerable heterogeneity. Nevertheless, some interesting principles have emerged in recent years (Kullmann, 2011). It appears that NMDAR-mediated pathway specificity is expressed in excitatory neurons but not in inhibitory neurons (Kumar and Huguenard, 2003), and NMDARs underlying excitatory inputs onto an inhibitory neuron are most likely homogenous (Maccaferri and Dingledine, 2002). Hence, there may be far more inhibitory neurons than excitatory neurons that can be classified as being distinct based solely on the type of NMDARs they express (Lei and McBain, 2002).

We report that memantine preferentially antagonizes NMDAR currents in PCs over Ints, as indicated by the IC_50_ values for these two types of neurons (PCs: absence of Mg^2+^ 7.7 μM, presence of Mg^2+^ 4.2 μM; Ints: absence of Mg^2+^ 17 μM, presence of Mg^2+^ 22.45 μM). Hill coefficients calculated in presence and virtual absence of Mg^2+^, from the sigmoid dose-response curves for these two types of neurons suggest that, in presence of Mg^2+^, PCs may possess at least two subtypes of NMDAR, one of which appears to have a higher affinity for memantine, whereas Ints may express a single NMDA receptor subtype with a lower affinity for memantine. In contrast, in the virtual absence of Mg^2+^, both PCs and Ints Hill coefficients, even if significantly different, suggest positive cooperativity. This discrepancy may be due to the fact that different types of NMDAR subtypes show different Mg^2+^ and voltage sensitivity.

In the hippocampus, PCs mainly express NR2A and NR2B subunits, while Ints have a larger percentage of NR2C and NR2D subunits (Monyer et al., 1994; Martina et al., 2003). We studied the molecular make-up of the native NMDARs using pharmacological tools and found that NMDARs in PCs contained ∼50% NR2A, ∼25% NR2B, and ∼25% NR2C-D, while Ints contained ∼20–25% NR2A, ∼35% NR2B, and ∼40–45% NR2C-D (Figure 7A), accounting for the different NMDAR kinetics observed in PCs and Ints. Very recently, it has been reported (Rauner and Köhr, 2011) that triheteromeric NR1/NR2A/NR2B receptors constitute the majority of the NMDAR population in adult rat hippocampus synapses and that antagonists such as Ro25-6981 are NR2B-directed but not NR1/NR2B-selective antagonists. In NR1/NR2A/NR2B receptors, the NR2B subunit slows deactivation kinetics, whereas the presence of NR2A confers voltage-dependence of decay. Consequently, a percentage of the NMDAR current reduction by Ro25-6981 that we observed could be due to antagonism at the NR1/NR2A/NR2B receptor, as well as at the NR1/NR2B receptor. Nevertheless, the differences in NMDAR kinetics in PCs and Ints reported in this study are attributable to neurons with different NMDAR subunit composition.

It has previously been proposed that memantine is relatively selective for NR2B. This is partially supported by the finding that memantine is three times more potent against NMDA-induced Ca^2+^ influx in human NR1a/NR2B receptors than in human NR1a/NR2A expressed in L(tk-) cells (Grimwood et al., 1996). However, these data are controversial, since different types of cells transfected with different types of NMDAR subunits showed different IC_50_ values: in oocytes NR2A 0.89 μM, NR2B 0.40 μM, NR2C 0.32 μM, and NR2D 0.28 μM (Parsons et al., 1999), and NR2A 0.29 μM, NR2B 0.23 μM (Avenet et al., 1997); in HEK-293 cells, NR2A 0.93 μM, NR2B 0.82 μM, and NR2D 0.47 μM (Bresink et al., 1996).

In this study, in PCs, in the presence of NVP-AAM077, memantine reduced NMDAR currents (NR2B, C-D: 50% of the total NMDAR current) by ∼42%, while in presence of R025-8169, memantine reduced the NMDAR currents (NR2A, C-D: 75% of the total NMDAR current) by ∼62%. Since, in the presence of NVP-AAM077 and Ro25-6981, memantine attenuated the residual NMDAR currents containing only the NR2C-D subunits by 28%, we conclude that in PCs memantine efficacy is NR2A > NR2B > NR2C-D. This is also true for Ints (Figure 7). We found the same effect of memantine on NMDARs containing NR2C-D in PCs and Ints. Consequently, we conclude that memantine antagonism depends on the molecular make-up of NMDARs expressed in different neuronal types.

At physiological Mg^2+^ concentrations (1 mM), it has been reported that memantine inhibition decreases nearly 20-fold at resting membrane potential for NR1/NR2A and NR1/NR2B receptors, while the effect on NR1/NR2C and NR1/NR2D decreases only threefold (Kotermanski and Johnson, 2009). However, in the hippocampal network at resting membrane potential the number of open NMDARs is extremely low (Martina et al., 2003). Indeed, the Mg^2+^ block of NR1/NR2A subunits and NR1/NR2B receptors is nearly complete at typical resting membrane voltages, and is relieved upon depolarization. PSCs are generated by glutamate liberated from the Shaffer collaterals and PSCs depend largely on the activation of AMPARs. AMPARs depolarize the post-synaptic membrane, causing the Mg^2+^ block to be relieved and NMDARs to open. Our experiments, in the presence of physiological Mg^2+^ (1 mM) at −30 mV and in the virtual absence of Mg^2+^ at −70 mV, show that in PCs, NMDARs have a higher affinity for memantine at −30 mV (Mg^2+^ block is relieved by depolarization; IC_50_ = 4.2 μM) than at −70 mV (IC_50_ = 7.7 μM; *p* = 0.03, non-parametric Wilcoxon Rank Test). In contrast, in Ints, NMDARs show no significant difference (*p* = 0.223, non-parametric Wilcoxon Rank Test) in the affinity for memantine at −70 mV (IC_50_ = 17 μM) and −30 mV (IC_50_ = 22.45 μM). These results further support our findings of variable antagonism of NMDARs in PCs and Ints under physiological stimulation. We suggest that the higher affinity of memantine for NMDARs in PCs is due to the higher percentage of NR2A in PCs compared to Ints. Clarke and Johnson (2008) demonstrated that the activity of NMDARs composed of NR1 and NR2B subunits (NR1/2B receptors) is enhanced by depolarization even in 0 Mg^2+^. Collectively, our findings and those of Kotermanski and Johnson (2009) support our hypothesis that memantine acts differently on NMDARs with different subunit composition. It has been shown that in freshly dissociated rat hippocampal neurons, memantine (10 μM) selectively antagonized NMDA (500 μM + glycine 5 μM) -induced inward currents in a voltage-dependent manner with an IC_50_ = 1.04 ± 0.26 μM (Parsons et al., 1996). This discrepancy with our data is probably due to the fact that here we recorded from only synaptic NMDAR activated by glutamate, while Parsons et al. (1996) activated synaptic, extra-synaptic, and somatic NMDARs using NMDA.

The glutamatergic system and, in particular, the NMDAR, plays an important role in the pathogenesis of numerous neurodegenerative disorders. However, the development of NMDAR antagonists as therapeutic agents has been disappointing because of side-effects caused by these drugs. Efforts have been directed toward low-affinity uncompetitive open-channel (use-dependent) blockers like memantine which are better suited for therapeutic intervention during excessive NMDAR activation, since their antagonism requires prior activation of the receptor. In a review of NMDAR pharmacology Parson and colleagues stated “too little activation is bad, too much is even worse” (Parsons et al., 2007). One approach to avoiding these caveats is to target specific NMDAR subtypes to restore balance between excitation and inhibition in the brain and maintain a therapeutic margin of safety. Although our studies were performed using juvenile rats, it is of value to consider extrapolation of these observations to memantine action on neuronal network activities in AD. The extracellular concentration of memantine in the brains of AD patients during clinical treatment has been estimated to be between 0.5 and 1 μM (Parsons et al., 2007). It may be therapeutically relevant to consider that, at these concentrations, our dose-response curve indicates that only NMDAR currents in PCs would be attenuated. It has been suggested that in animal models of AD, the excitation-inhibition balance in hippocampal circuitry is shifted toward over-excitation (Schmitt, 2005), and that dysfunction of inhibitory Ints are likely to increase synchrony among excitatory principal cells and contributes to the destabilization of neuronal networks (Palop and Mucke, 2010). At a concentration of 1 μM, memantine would reduce PC excitation of the hippocampal network without attenuating Int inhibition (Figure 7B). However, at higher concentrations (>10 μM), the antagonism of memantine on PCs and Ints is similar, conferring no beneficial effect of memantine on the excitatory-inhibitory balance.

It has been hypothesized that memantine is selective for extra-synaptic NMDAR receptors (Chen et al., 1998; Lipton, 2007; Xia et al., 2010). Extra-synaptic receptors may become activated when glutamate spills out of the synapse during prolonged depolarization or when glutamate transporters reverse operation (Jabaudon et al., 2000). However, it has also been suggested that the most important determinant of memantine selectivity – synaptic vs. extra-synaptic – is not dependent on the NMDAR localization but on the pattern of receptor activation (Wroge et al., 2012). Wroge et al. (2012) suggest that, at low memantine concentrations, transient receptor activation during brief transmitter presence limits the amount of memantine channel block during synaptic activation. By contrast, sustained activation of receptors facilitates memantine inhibition (Wroge et al., 2012). Moreover, Danysz and Parsons (2012) suggest that the most important factors for the action of memantine on synaptic vs. extra-synaptic receptor is the resting membrane potential of the neuron and the NMDA ligand and voltage gating properties. They suggest that the NMDAR voltage dependency can be a burden in chronic disease states such as those occurring in AD. Factors that disturb the normal resting membrane potential of neurons can have severe impact on the normal function of NMDA receptors as these can lead to a tonic relief of their voltage-dependent modulation by Mg^2+^ (Danysz and Parsons, 2012). In this context, our quantification of the memantine effects on synaptic evoked NMDAR current in the two main neuronal populations (glutamatergic and GABAergic) is important to future consideration of *in vitro* assessment of pharmacological efficacy and relevance to physiological or pathophysiological situations.

## Conflict of Interest Statement

The authors declare that the research was conducted in the absence of any commercial or financial relationships that could be construed as a potential conflict of interest.

## Author Note

Research conception and design: Marzia Martina, Data acquisition: Marzia Martina and Tanya Comas, Analysis data: Marzia Martina, Wrote or contributed to the writing of the manuscript: Marzia Martina and Geoffrey Mealing.
